# Peculiarities of Fatigue Crack Growth in Steel 17H1S after Long-Term Operations on a Gas Pipeline

**DOI:** 10.3390/ma16082964

**Published:** 2023-04-07

**Authors:** Volodymyr Vira, Halyna Krechkovska, Volodymyr Kulyk, Zoia Duriagina, Oleksandra Student, Bogdan Vasyliv, Veronika Cherkes, Tetiana Loskutova

**Affiliations:** 1 Department of Strength of Materials and Structural Mechanics, Lviv Polytechnic National University, 12 S. Bandera Str., 79013 Lviv, Ukraine; 2 Department of Diagnostics of Materials Corrosion-Hydrogen Degradation, Karpenko Physico-Mechanical Institute, 5 Naukova Str., 79060 Lviv, Ukraine; 3 Department of Materials Science and Engineering, Lviv Polytechnic National University, 12 S. Bandera Str., 79013 Lviv, Ukraine; 4 Department of Materials Engineering, The John Paul II Catholic University of Lublin, 14 Racławickie Al., 20-950 Lublin, Poland; 5 Department of Hydrogen Technologies and Alternative Energy Materials, Karpenko Physico-Mechanical Institute, 5 Naukova Str., 79060 Lviv, Ukraine; 6 Department of Building Production, Lviv Polytechnic National University, 12 S. Bandera Str., 79013 Lviv, Ukraine; 7 Department of Materials Science and Heat Treatment, National Technical University of Ukraine “Igor Sikorsky Kyiv Polytechnic Institute”, 03056 Kyiv, Ukraine

**Keywords:** main gas pipeline steels, microstructure, fatigue crack growth resistance, R-ratio, Paris’ law region, fracture micromechanism

## Abstract

This work presents the results of metallographic studies and the tensile, impact, and fatigue crack growth (FCG) resistance tests of 17H1S main gas pipeline steel in the as-received (AR) state and after a long-term operation (LTO). A significant number of non-metallic inclusions forming chains stretched along the direction of pipe rolling were found in the microstructure of the LTO steel. The lowest values of elongation at break and impact toughness of the steel were determined for the lower part of the pipe close to its inner surface. FCG tests at a low stress ratio (R = 0.1) did not reveal a significant change in its growth rate in degraded 17H1S steel compared to steel in the AR state. During tests at a stress ratio R = 0.5, the effect of degradation was more pronounced. The Paris’ law region of the da/dN—∆K diagram for the LTO steel corresponding to the lower part of the pipe close to its inner surface was higher than those for the steel in the AR state and the LTO steel corresponding to the higher part of the pipe. Fractographically, a significant number of delaminations of non-metallic inclusions from the matrix were recognized. Their role in the embrittlement of steel, especially steel from the lower part of the pipe close to its inner surface, was noted.

## 1. Introduction

The long-term operation (LTO) of main gas pipelines causes the degradation of the metal of pipes, which reduces their efficiency and increases the risk of unexpected breakdowns. The specificity of the main gas pipeline operation is related to the combined long-term impact on pipe components of operational stresses (due to gas pressure in pipes, bending moments caused by landslides, thermal stresses caused by seasonal temperature changes, etc.) and corrosive environments (soil water, bottom water, or condensate). An analysis of the causes of gas pipeline accidents confirmed that more than half of them occurred as a result of the combined effect of corrosive-active environments and operational loads [1,2,3,4,5]. The main reasons for the loss of performance of main gas pipelines include metal corrosion on the outer surface of the pipes (due to a violation of their anti-corrosion protection) or on their inner surface, which is associated with the transportation of hydrocarbons with a significant content of highly aggressive impurities [6]. Such conditions of their long-term operation cause a change in the mechanical properties of steels, particularly strength, plasticity, and resistance to brittle fracture [7,8,9,10]. Scattered damage, which is intensively analyzed by researchers and operators, is also a feature of the degradation of objects operated in climatic conditions. In particular, it was shown in studies [10,11,12,13] that the thirty-year operation of the main oil pipeline and the oil storage tank had a negative effect not only on the mechanical properties of steels but also on their electrochemical and corrosion characteristics [14,15]. At the same time, the metal of those sections of structures that were constantly in contact with the corrosive-hydrogenating environment in the form of bottom water degraded the most [16]. Such an environment, as one of the important factors of the operational degradation of pipe components of the pipeline system, contributes to the formation and adsorption of hydrogen on the metal surface with its further penetration into the depth of the pipes. Corrosion damage is also detected on the inner surfaces of the pipes, which proves the importance of taking into account the aggressive influence of corrosive-active components of the environment, which is unavoidable during the transportation of hydrocarbons. The appearance of corrosion damage is associated with the formation of water condensate inside the pipes [17]. Such conditions of the operation of gas pipeline steels lead to their tendency toward corrosion cracking under stress as one of the frequent causes of pipeline failures [10,18].

It is also noted that gas pipelines can often be subjected to cyclic loading (for example, due to temperature changes in the above-ground sections of pipelines or the operation of gas pumping equipment) [19]. Fatigue microcracks most often arise from corrosion pits as stress concentrators and later grow into macrocracks that advance into the depth of the pipes. The character of crack growth depends on several operational factors [20]. The stress intensity factor (SIF) at the tip of the cracks in the pipes, the frequency, stress ratio, and shape of the loading cycles are among the determining mechanical factors influencing the rate of FCG in the pipes. Of course, this rate also depends on the testing temperature and corrosive activity of the operating environment. The character and intensity of the influence of the environment, in turn, also depend on the “material-environment” system and the susceptibility of the steel of the structural components relative to the influence of hydrogen on the rate of FCG in them [21].

It is known that predicting the fatigue life of structural elements is impossible without taking into account the presence of defects in materials [22,23]. The weak link theory (WLT) was considered, taking into account the size and location of defects. It was believed that one of the most important factors affecting the fatigue strength of a material is a change in the volume size factor. Using the proposed approach, the authors determined the size of a critical defect that causes the initiation of a fatigue crack, which significantly worsens the fatigue properties of the material. This model has been improved by combining WLT with the concept of strain energy [23]. The authors determined the area of critical damage based on the strain energy, which is exhaustive for the analysis of the local distribution of elastoplastic stresses/strains in the vicinity of the defect. The process of the development of fatigue damage in the Sn-3.0Ag-0.5Cu (SAC305) alloy was explained by the generation of entropy [24]. The energy dissipated under the fatigue loading of the evolving β-Sn dendritic phase and the surrounding Sn-Ag-Cu ternary eutectic network is the basis of their explanations. The analyzed approaches are interesting and can also be useful for predicting fatigue life, including pipeline steels.

Increasing the crack growth resistance of the gas pipe material (especially in the stage preceding the spontaneous propagation of cracks in the gas pipe components, when the depth of damage does not exceed 40% of the nominal pipe wall thickness, and the maximum crack length is less than the critical length calculated for an examined pipe according to the criteria of fracture mechanics) gives time to detect sub-critical damage and repair pipelines [25].

Therefore, the combined long-term effect of static, cyclic, and dynamic loads and corrosive environments (from water-soluble corrosive-active components of the transported product or groundwater) on the main gas pipeline metal during its operation causes the initiation and propagation of crack-like defects and corrosion-mechanical cracks, which, reaching critical dimensions, lead to a violation of the integrity of gas pipelines.

This work is aimed at studying the impact of 17H1S main gas pipeline steel degradation after LTO on the FCG rate at a high stress ratio by taking into account the positioning of the cut specimens relative to the positioning of the pipe and identifying microstructural and fractographic features of steel degradation.

## 2. Materials and Methods

The 17H1S main gas pipeline steel was studied in the as-received (AR) state and after LTO for 31 years. In the first case, specimens were cut from a stock pipe. In both cases, the pipe wall thickness was 10 mm. The chemical composition of steel from both pipes corresponded to the regulated composition for this steel (Table 1).

**Figure 1:**
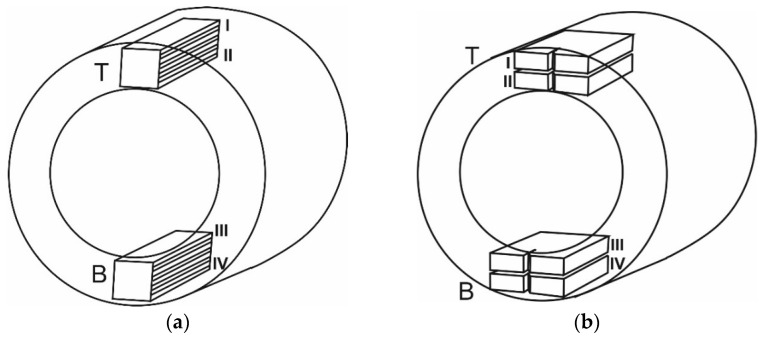
Schemes of cutting specimens from pipes for (**a**) the metallographic analysis of steel and (**b**) FCG resistance (mechanical) tests. Zones of cutting specimens from the upper (referred to as T) and lower (B) parts of the pipe in the positions close to its outer (zones I and IV) and inner (zones II and III) surfaces are marked.

The microstructure and mechanical properties of the steel were analyzed on specimens cut from the pipes in corresponding zones (Figure 1). The specimens of the steel in the AR state were cut from the stock pipe close to its outer (zone I) and inner (zone II) surfaces. The specimens of the steel after LTO were cut from the upper (referred to as T) and lower (B) parts of the corresponding pipe in the positions close to its outer (zones I and IV) and inner (zones II and III) surfaces.

**Figure 2:**
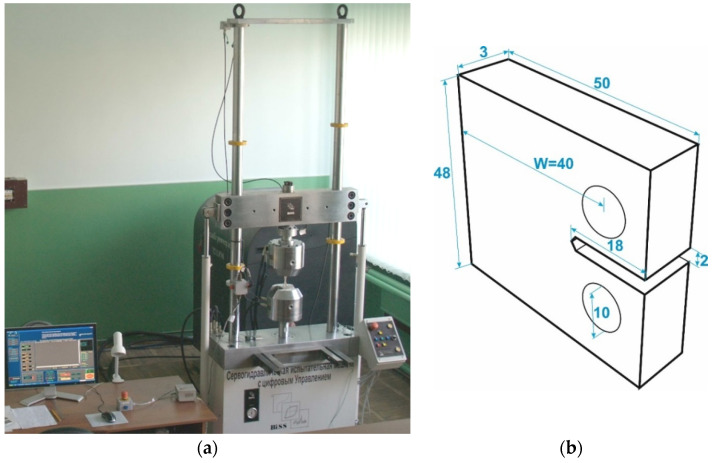
BISS servo-hydraulic testing machine (**a**) and a compact specimen (**b**) for FCG resistance tests. Dimensions are given in mm.

Fatigue tests were performed on a 50 kN BISS (BISS, Bangalore, India) servo-hydraulic testing machine (Figure 2a). A compact specimen with a V-shaped stress concentrator (Figure 2b), oriented in the direction of pipe rolling (made in a plane parallel to the direction of pipe rolling) (Figure 1b), was used for research. The test was carried out at a constant amplitude of the load with a frequency of 10 Hz and the values of the R = P_min_/P_max_ stress ratio of 0.1 and 0.5 in air and at room temperature. The length of the fatigue macrocrack and the moment of its initiation were recorded with a KM-6 cathetometer at a 25-fold magnification with a measurement error of ±0.02 mm.

The microstructure of steels was studied in the axial sections of the pipes (Figure 1a). For its detection, multiple alternating polishing and etching operations of the specimens with a 2–4% solution of nitric acid in alcohol were used. Metallographic studies of the steels in the AR state and after the operation, as well as the fractographic features of crack growth during FCG resistance tests, were analyzed using a scanning electron microscope (SEM) EVO-40XVP (Zeiss, Oberkochen, Germany).

To evaluate the technical state of the studied steels, 3 mm thick flat specimens were tested in the air under uniaxial tension at a deformation rate of 3.3 ∙ 10^−3^ s^−1^. The test results were used to determine the characteristics of strength (ultimate tensile strength σ_UTS_ and yield strength σ_YS_) and plasticity (elongation at break EB). The Charpy impact test was performed on standard transverse Charpy V-notch specimens with a notch tip radius of 0.25 mm to determine the values of impact toughness KCV. The direction of specimen fracture occurred in the direction of pipe rolling.

## 3. Results

### 3.1. Metallographic Studies

**Figure 3:**
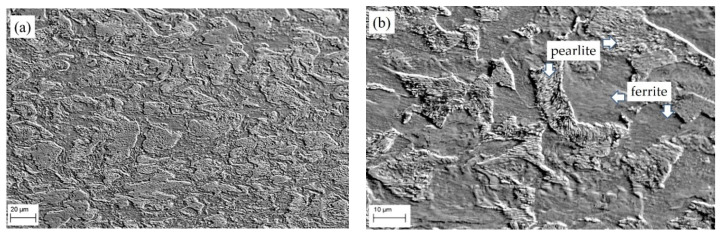
SEM microstructure of 17H1S steel in the AR state at (**a**) low and (**b**) high magnifications.

The microstructure of the 17H1S main gas pipeline steel in the AR state and after LTO was analyzed. It was found that in the AR state, 17H1S steel had a typical ferrite-pearlite structure with rather small cementite plates within them (Figure 3).

**Figure 4:**
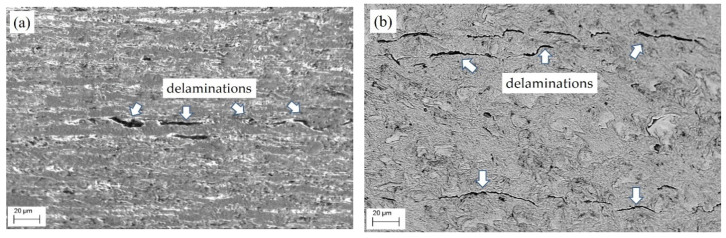
SEM microstructure of the LTO steel cut from (**a**) the upper part of the pipe (position T in Figure 1a) and (**b**) its lower part (position B in Figure 1a) close to its inner surface (zones II and III in Figure 1a). Delaminations are indicated by arrows.

The operating environment, as a rule, negatively affects the performance of critical structures such as gas pipelines. Moreover, the state of the metal changes primarily near the surface of the pipe, which is in contact with the environment. The microstructural features of the LTO steel in the sections located during operations in the lower (position B in Figure 1a) and upper (position T) parts of the pipe close to its inner surface (zones II and III in Figure 1a) were analyzed. As a result of the LTO steel, a significant number of defects in the form of delaminations parallel to the pipe axis were found in its structure (Figure 4). Moreover, the microstructural analysis of the LTO pipe showed that such delaminations in specimens cut from its lower part were longer than those of the upper part and in some places reached 70 μm.

In addition, there is an obvious difference in the microstructure of the analyzed parts (T and B) of the LTO steel. In the upper part, a clear texture of ferrite and pearlite grains was preserved (Figure 4a). In contrast, in the microstructure of the steel from the lower part of the pipe, the only delaminations stretched along the rolling direction indicated the possible presence of a texture at the beginning of the operation (Figure 4b). In addition, the opening of delaminations in the microstructure of the upper part of the pipe was much greater than that in the microstructure of its lower part. This is a sign of a greater margin of plasticity of the surrounding matrix in the first case compared to the second one, which is consistent with the data on their mechanical properties.

**Figure 5:**
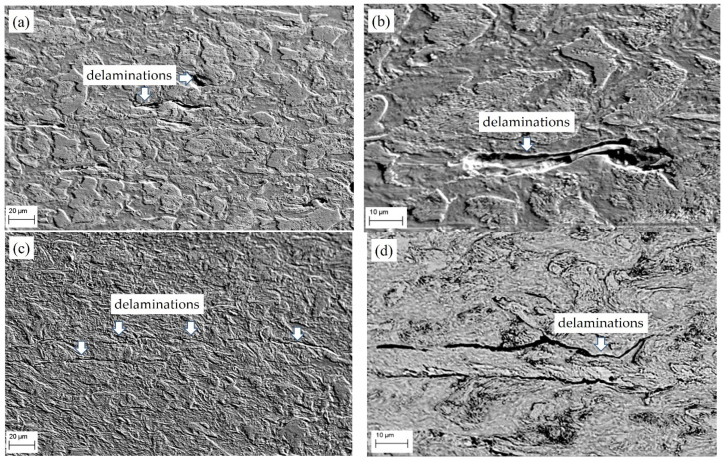
SEM microstructure of the LTO steel cut from (**a**,**b**) the upper part of the pipe (position T, zone II in Figure 1a) and (**c**,**d**) its lower part (position B, zone III in Figure 1a) close to its inner surface. Delaminations are indicated by arrows.

At a higher magnification, the described microstructure features of the LTO steel for both the upper and lower parts of the pipe are even more obvious (Figure 5). It is assumed that the identified delaminations formed in the steel during its operation under the influence of operating stresses and hydrogen absorbed by the metal. They formed parallel to the rolling direction in the metal layer with weak inclusion adhesion to the matrix (Figure 5). In operated steel, non-metallic inclusions were found in the form of mostly round oxides of various compositions (up to 7 microns in diameter) and manganese sulfides elongated in the rolling direction, the average size of which did not exceed 40 microns. Delaminations in steels occur for various reasons related to the anisotropy of the microstructure (different texture, arrangement of non-metallic inclusions in the matrix parallel to the rolling direction, and intergranular fracture along the boundaries of former austenite grains) [26]. The arrangement of non-metallic inclusions elongated in the rolling direction in the form of long chains contributes to the decohesion of these inclusions (Figure 5b). Such inclusions can serve as traps for the accumulation of hydrogen. In studies [27,28], it is described that atomic hydrogen can accumulate in the formed voids and recombine into the molecular state, creating pressure in them, which facilitates hydrogen-initiated failure due to the reduction in cohesion between atoms in the metal. Hydrogen delamination is typical for gas pipelines since the pipe manufacturing technology involves metal rolling and, accordingly, the elongation of non-metallic inclusions with the weakening of their adhesion to the matrix. On the other hand, the hydrogenation of the pipe wall, which is possible from the side of its inner surface [17], also contributes to the accumulation of molecular hydrogen in the defects formed at the interphase boundaries between the inclusions and the matrix, achieving high pressures in them. This contributes to the development of microdamage scattered throughout the volume of the metal and the deterioration of the mechanical properties of steel, especially their plasticity and resistance to brittle fractures [29,30,31].

**Figure 6:**
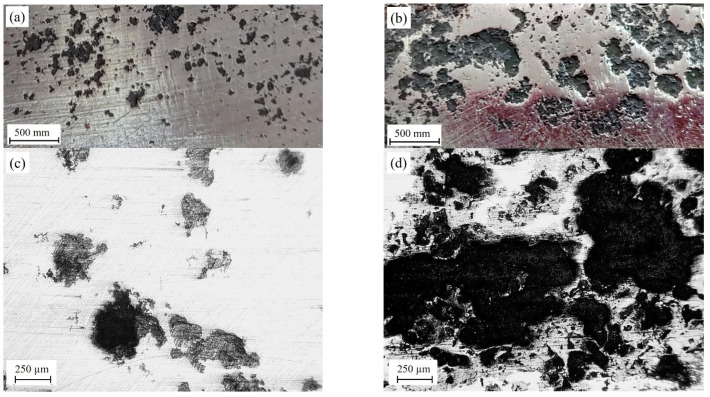
Corrosive damage to the inner surface of the LTO pipe corresponding to (**a**,**c**) the upper part of the pipe and (**b**,**d**) its lower part.

The inner surface of the LTO pipe in its upper and lower parts (zones II and III in Figure 1b, respectively) was cleaned by the ultrasonic method. Different grades of corrosion damage found on the surfaces of these parts indicate a significant difference in the intensity of the aggressive effect of the corrosive environment on the metal of the pipe during operations. Much less surface damage was found in the upper part of the pipe (Figure 6a,c) than in the lower one (Figure 6b,d). This is an obvious feature of more intense damage to the metal on the side of the inner surface of the lower part of the pipe during the LTO of a gas pipeline.

### 3.2. Mechanical Properties of 17H1S Steel Estimated under Tension and Impact

To examine the technical condition of the main gas pipeline steel after LTO, the characteristics of strength and plasticity and impact toughness regulated by industry documents were used. The obtained mechanical characteristics of 17H1S steel in the AR state and after LTO are shown in Table 2. In general, the strength characteristics of the steel in the AR state, obtained in tensile tests of specimens cut from the pipe close to the outer and inner surfaces, satisfied the regulated values for 17H1S steel (σ_UTS_ ≥ 510 MPa and σ_YS_ ≥ 363 MPa).

After the LTO of the main gas pipeline steel, its ultimate tensile strength σ_UTS_ slightly decreased but remained within the permissible limits (except for the value of σ_UTS_ = 500 MPa obtained on specimens from the upper part of the pipe close to its outer surface, which fell below the permissible value). Meanwhile, the values of yield strength σ_YS_ for the LTO steel became lower than the regulated value (σ_YS_ = 363 MPa). Moreover, regardless of the pipe part that the test specimens were cut from (the upper or lower part of the LTO pipe of the gas pipeline), the values of σ_YS_ determined from the specimens cut close to the outer surface of the pipe were lower than those corresponding to the material close to its inner surface. Therefore, the negative effect of operational degradation of the steel was more pronounced in the lower part of the pipe. The scheme for the development of the gas pipeline steel degradation proposed in studies [32,33] was used to estimate a degradation stage for the studied steel. Since both the strength characteristics (especially the yield strength) of the LTO steel decreased, this was evaluated according to the mentioned scheme as the stage of damage coalescence that occurred in the steel during operation.

Despite the expected decrease in the values of elongation at the break of the LTO steel compared to the steel in the AR state, it was also noted that in the operated steel value of elongation at break was slightly higher for specimens cut from the pipe close to its outer surface than that recorded for the material close to the inner surface. It was also noted in contrast to the lowest values of the strength characteristics obtained for the LTO steel from the upper part of the pipe close to its outer surface, and its elongation value was the highest. This is a typical change in these characteristics. In general, the metal located close to the inner surface of the pipe part, which during the operation was situated at the bottom and could come into contact with condensate and corrosive-active impurities dissolved in it, which are usually present in the transported gas, was estimated in terms of mechanical properties under tension as the most degraded.

Resistance to the brittle fracture of materials is considered one of the most sensitive indicators of steel degradation. It is used to substantiate steel workability in components of various structures [34,35,36,37,38,39,40,41,42]. On the one hand, this indicator characterizes the ability of the material to fail by a ductile mechanism with clear signs of plasticity, and on the other hand, it characterizes the ability of the material to resist the most dangerous, almost deformation-free fracture by the mechanism of brittle cleavage, which is especially important to consider for steels operated at subzero temperatures. The long-term practice of operating structures shows that steels in different structural states are characterized by different susceptibilities to brittle failure, which largely determines their lifetime [9,43,44,45,46]. With this in mind, study [8] estimated the impact toughness of 17H1S steel in the AR state and after LTO in the temperature range from +20 to 60 °C. It was noted that the steel from the lower part of the pipe close to its inner surface turned out to be the most degraded in terms of impact toughness at a temperature of +20 °C (Table 2). Generally, the LTO steel showed a lowered impact toughness. Even in the case of the steel from the upper part of the pipe close to its outer surface showing the lowest strength and highest elongation, its impact toughness was low.

### 3.3. The Results of the FCG Resistance Test of 17H1S Steel

Based on the FCG resistance test at a load frequency of 10 Hz and two values of the stress ratio (R = 0.1 and 0.5), the corresponding regularities of a change in the FCG rate da/dN depending on the stress intensity factor (SIF) range ∆K for steel in the AR state and after LTO were analyzed. During the analysis, the impact of two factors was singled out, namely a mechanical factor caused by a change in the static component in the loading cycle due to the increase in R and a microstructural one caused by the degradation of steel during the LTO of a gas pipeline. In addition, when analyzing steel degradation, the relative location of the examined pipe metal in two different places during gas pipeline operation (the upper (T) or lower (B) parts, close to the outer ((I) and (IV)) or inner ((II) and (III)) surfaces of the pipe) was taken into account (notation in Figure 1).

**Figure 7:**
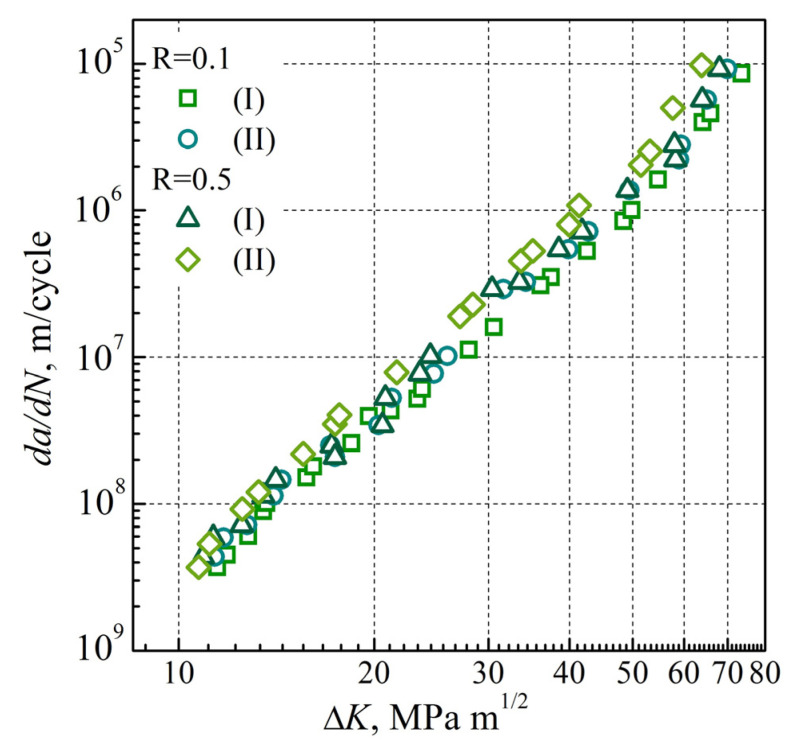
Dependences of the FCG rate on the SIF range (*da*/*dN*—∆*K*) obtained for 17H1S steel in the AR state at values of the stress ratio of 0.1 ((I),(II)) and 0.5 ((I),(II)) on specimens cut from the stock pipe close to its outer (I) and inner (II) surfaces.

By analyzing the Paris’ law regions in the da/dN—∆K dependences obtained for 17H1S steel in the AR state (Figure 7), almost no impact of the stress ratio on da/dN was revealed for the metal cut from the pipe close to its outer or inner surfaces. All the obtained data formed a single narrow data scatter band, which indicated that at the corresponding levels of ∆K, almost no impacts of both stress ratio R and relative location of the examined pipe metal on the crack growth rate da/dN exist. The insensitivity of crack growth resistance characteristics relative to steel degradation in the Paris’ law region is consistent with the conclusion of other authors, particularly [32,47]. A slight deviation of the da/dN—∆K diagrams from linearity was only observed for the steel in the AR state tested at R = 0.5 when approaching the crack growth rate relative to that corresponding to the critical stress intensity factor range ∆*K_fc_*, which quantitatively characterizes the fracture toughness of the material.

**Figure 8:**
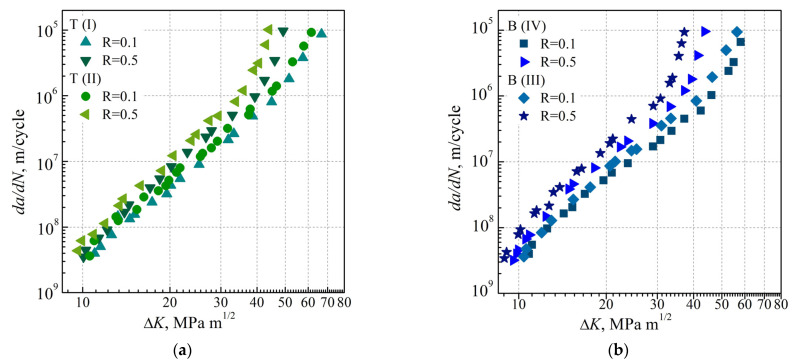
Dependences of the FCG rate on the SIF range (*da*/*dN*—∆*K*) obtained for 17H1S steel at values of the stress ratio of 0.1 and 0.5. Specimens of the steel were cut from (**a**) the upper T and (**b**) lower B parts of the LTO pipe in positions close to its outer ((I),(IV)) and inner ((II),(III)) surfaces.

In the case of testing the LTO steel, the impact of the stress ratio manifested itself much more clearly in the form of an increase in the crack growth rate even in the Paris’ law region (Figure 8). It was detected both in the metal of the upper part of the pipe (Figure 8a) and to a much greater extent in the metal of its lower part (Figure 8b). On this basis, it was concluded that the FCG rate to a certain extent still correlates to the gradient of microstructural changes both in the transverse direction of the pipe (i.e., normal to its axis) and in the longitudinal direction (i.e., parallel to its axis in its upper or lower part). Moreover, the impact of the stress ratio on the FCG rate da/dN was the strongest in specimens cut from the lower part of the pipe close to its inner surface.

**Figure 9:**
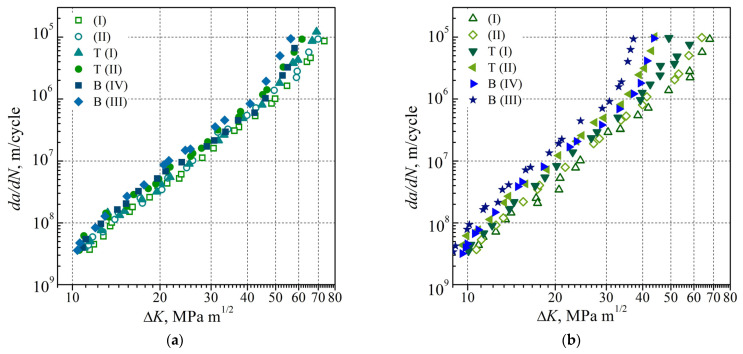
Dependences of the FCG rate on the SIF range (*da*/*dN*—∆*K*) obtained for 17H1S steel in the AR state (light symbols) and after LTO at values of the stress ratio of (**a**) 0.1 and (**b**) 0.5. Specimens of the steel in the AR state were cut from the stock pipe close to its outer (I) and inner (II) surfaces. Specimens of the LTO steel were cut from the upper (T (I), T (II)) and lower (B (III), B (IV)) parts of the corresponding pipe in the positions close to its outer ((I), (IV)) and inner ((II), (III)) surfaces.

Given the strongest effect of the stress ratio found for specimens cut from the lower part of the pipe close to its inner surface, the degradation effects in the metal cut from the upper and lower parts of the LTO pipe were also compared. To carry this out, corresponding da/dN—∆K diagrams constructed for steel in the AR state and after LTO under equivalent conditions in terms of the stress ratio and relative location of the examined pipe metal were compared (Figure 9). The effect of the material degradation on the FCG rate da/dN was found to be significantly weaker at stress ratio R = 0.1 (Figure 9a) compared to that observed at stress ratio R = 0.5 (Figure 9b). Moreover, the maximum negative impact of the material degradation was most clearly manifested in the metal cut from the lower part of the pipe close to its inner surface (Figure 9b). A higher crack growth rate in operationally degraded steel was observed not only at high values of the FCG rate da/dN but also almost from the beginning of the Paris’ law region. Therefore, it was believed that the fractographic features of crack growth in operationally degraded steel should be analyzed first on the fracture surfaces of the specimens cut from a pipe part close to its inner surface; then, it underwent the FCG resistance test under a higher stress ratio.

Such assumptions can be confirmed using an approach that takes into account the location and size of defects [22,23]. Namely, the concept of strain energy can be useful for determining the area of critical damage [23]. This would make it possible to more reasonably explain the increase in the crack growth rate in operationally degraded steel practically from the beginning of the Paris’ law region on the da/dN—ΔK diagrams.

At the same time, it was believed that an increased stress ratio will be an additional factor that will facilitate the visualization of degradation features and the possible embrittlement of the LTO steel.

### 3.4. Microfractographic Analysis of Damage Features of 17H1S Gas Pipeline Steel after LTO

Fracture surfaces of steel specimens cut from both the pipe in the AR state (zone II in Figure 1b) and the LTO pipe (zones II and III in Figure 1b) were subjected to a fractographic analysis after the FCG resistance test. Specimens tested at R = 0.5 were chosen for the principal fractographic analysis, whereas specimens tested at R = 0.1 were chosen only for comparative analyses. Special attention was paid to the fracture surfaces of specimens cut from the LTO pipe, as the discrepancy of the FCG rate (at the same ΔK levels) was most clearly observed.

**Figure 10:**
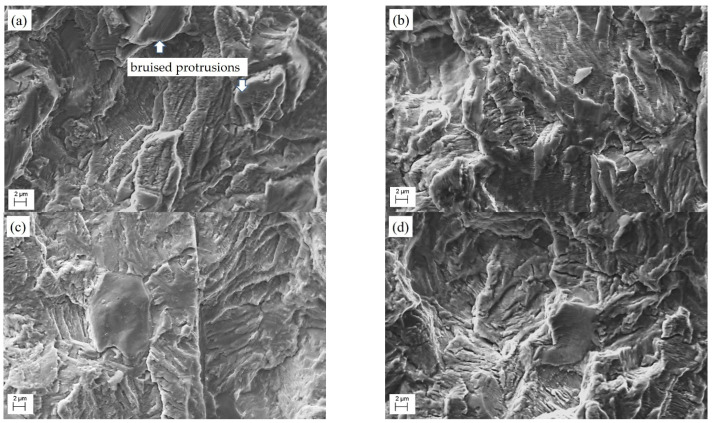
SEM fractography of specimens of 17H1S steel (**a,b**) in the AR state and (**c**,**d**) after LTO underwent the FCG resistance test at a frequency of 10 Hz and values of the stress ratio of (**a**,**c**) 0.1 and (**b**,**d**) 0.5. The SEM images correspond to the ΔK level of about 23–25 MPa m^1/2^.

Traces of the contact of the adjacent fracture surfaces in the unloading half-cycle were observed on the fracture surfaces of specimens tested at R = 0.1 for both the steel in the AR state (Figure 10a,b) and the LTO one (Figure 10c,d). They were in the form of worn small microstructure components and bruised protrusions (Figure 10a,c), thus greatly complicating the detection of differences between fracture surfaces. Small microstructure components could only be detected in the concavities on fracture surfaces, where they were protected by the ridges bordering them. At the same time, the ridges themselves were significantly damaged and appeared on the fractograms as bright areas. At a higher stress ratio value (R = 0.5), the number of such damaged ridges on the fracture surfaces was significantly smaller (Figure 10b,d), which is due to a greater opening of the crack banks in the unloading half-cycle (compared to that at a lower stress ratio value) and, accordingly, a decrease in the possibility of contacting the banks. Therefore, less damaged fracture surfaces of specimens tested under the higher stress ratio value were chosen to search for fracture features associated with steel degradation under operational conditions.

**Figure 11:**
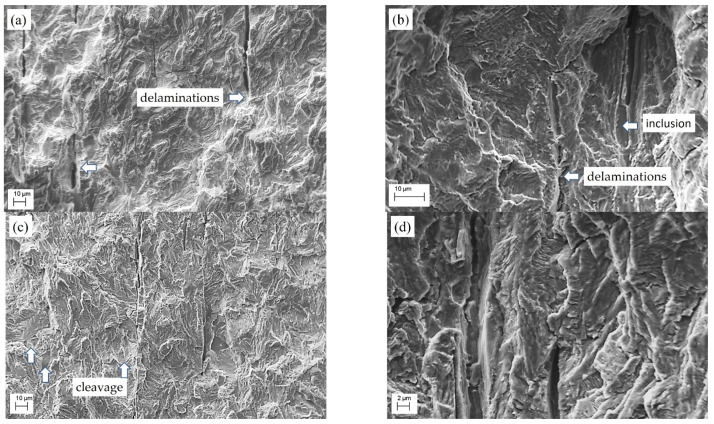

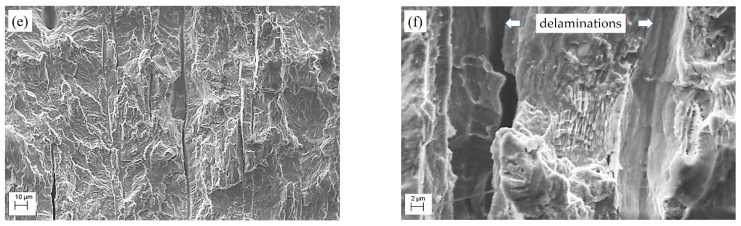
SEM fractography of specimens of 17H1S steel (**a**,**b**) in the AR state and (**c**–**f**) after LTO cut from the upper (**c**,**d**) and lower (**e**,**f**) parts of the corresponding pipe in the positions close to its inner surface and undergoing the FCG resistance test at a frequency of 10 Hz and a stress ratio of 0.5. The SEM images correspond to the ΔK level of about 32–35 MPa m^1/2^. Cleavage, inclusion and delaminations are indicated by arrows.

**Figure 12:**
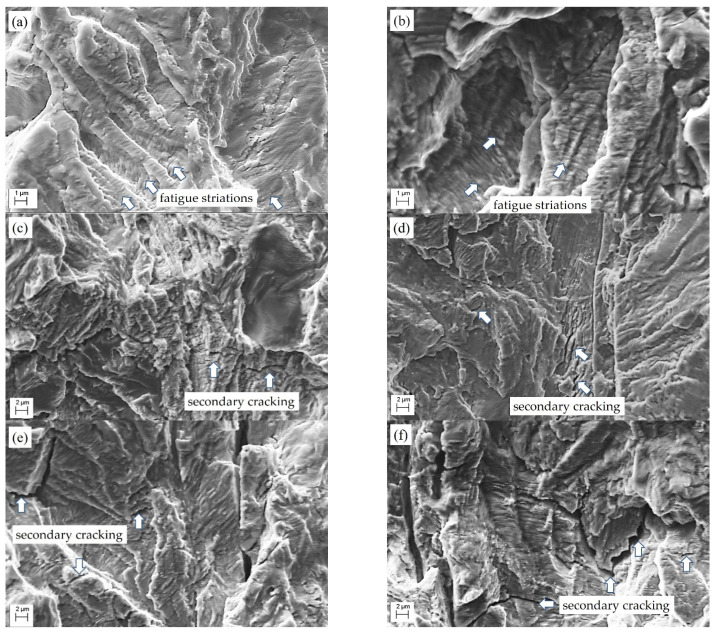
SEM fractography of specimens of 17H1S steel (**a**,**b**) in the AR state and (**c**–**f**) after LTO cut from the upper (**c**,**d**) and lower (**e**,**f**) parts of the corresponding pipe in the positions close to its inner surface and undergoing the FCG resistance test at a frequency of 10 Hz and a stress ratio of 0.5. The SEM images correspond to the ΔK levels of about (**a**,**c**,**f**) 15–20 MPa m^1/2^ and (**b**,**d**,**f**) 40–45 MPa m^1/2^. Fatigue striations and secondary cracking are indicated by arrows.

The features of the fatigue fracture surfaces of 17H1S steel specimens cut from both the pipe in the AR state and the LTO pipe in the positions mentioned above and also undergoing the FCG resistance test at R = 0.5 were analyzed because the highest crack growth rate was obtained for these specimens (Figure 11 and Figure 12).

The large number of delaminations found on the fracture surfaces of specimens of LTO steel can be considered the first feature of steel degradation. Moreover, their number on the fracture surfaces of specimens cut from the upper part of the LTO pipe (zone II in Figure 1b) was smaller (Figure 11c,d) compared to that detected for the lower part (zone III in Figure 1b) of the pipe (Figure 11e,f). Delaminations were also detected on the fracture surface of a specimen of the steel in the AR state (Figure 11a,b). However, firstly, they were rather an exception to the rule, and secondly, they were located in a fracture surface area corresponding to a high crack growth rate, where crack growth was accompanied by significant plastic deformation with respect to the metal in the process zone. Therefore, delaminations were formed by a ductile mechanism, which was evidenced by their lenticular shape with the significant opening of their banks. In contrast, in the LTO steel, inclusions delaminated from the matrix usually served as nucleation centers for brittle cleavage in local areas (Figure 11c,d), or even the growth of multiple fatigue cracks was initiated by delaminations in a plane perpendicular to the delamination planes (Figure 11f). Moreover, these local cracks advanced normally to the plane of the main fatigue crack. Therefore, the stress concentration around the delaminations (Figure 11f) was sufficient for the initiation of a fatigue crack from one of them and merged with the nearest delamination. Most likely, it happened in front of the main crack tip. As the crack front advanced, these local delamination-initiated damages merged with the main crack, causing the crack to grow in spurts. The highest FCG rate (at the extreme for ΔK more than 30 MPa m^1/2^) determined on the specimen from the lower part of the pipe close to its inner surface was found to correlate to the described features of steel degradation, as their number was the greatest.

A typical fatigue relief of fracture surfaces was revealed for areas corresponding to low crack growth rates, i.e., the beginning of the Paris’ law region, in SEM images taken at higher magnification (Figure 12). In these areas, the festoons that were formed along the direction of crack growth were covered across by parallel rows of fatigue striations. Secondary cracking, which decorated the striations, was noted as a feature of steel degradation (Figure 12c–f). Moreover, the higher the crack growth rate (the higher the level of ΔK), the more distinctly this feature manifested. In general, the fracture surface of a specimen of steel in the AR state was divided into small festoons, in which prerequisites were created for crack propagation in a certain direction, and the membranes between them failed due to stretching with significant plastic deformation (Figure 12a,b). In contrast, the festoons formed in specimens of the LTO steel were usually much wider, and the gaps between them were smaller, which was interpreted as the embrittlement of the steel due to degradation (Figure 12c–f). As a result, the height of the fatigue fracture surface relief in the LTO steel decreased.

Summarizing the results of fractographic studies, we concluded that even minor changes in the fatigue crack growth rate are manifested by embrittlement elements on the fracture surfaces of the specimens of the LTO steel.

## 4. Conclusions

The results of tensile and impact tests of 17H1S gas pipeline steel after the long-term operation confirmed its weakening with a simultaneous decrease in plasticity and impact toughness characteristics. The minimum values of elongation at break and impact toughness corresponded to the steel located in the lower part of the long-term operated pipe close to its inner surface.

In the microstructure of 17H1S steel, chains of non-metallic inclusions positioned parallel to the direction of pipe rolling were revealed. In the long-term operated steel, their visualization was easier due to their separation from the matrix (decohesion process) with the formation of large longitudinal delaminations.

The fatigue crack growth resistance tests of steel specimens at a low stress ratio (R = 0.1) revealed a slight change in the crack growth rate in 17H1S operationally degraded steel compared to the steel in the as-received state. Tests at R = 0.5 showed that the effect of degradation was more pronounced. The Paris’ law region of the da/dN—∆K diagram for the long-term operated steel corresponding to the lower part of the pipe close to its inner surface was higher than those for the steel in the as-received state and the long-term operated steel corresponding to the higher part of the pipe.

On the fracture surfaces of specimens of the operated 17H1S steel, a significant number of delaminations of non-metallic inclusions from the matrix, elongated in the direction of fatigue crack growth, were revealed. Their influence was most pronounced on the fracture surface of the steel in the lower part of the pipe close to its inner surface. Secondary cracks, which decorated the fatigue striations, and a decrease in the height of the fatigue fracture relief were also recognized to be the features of degradation of the long-term operated steel.

## Figures and Tables

**Table 1:** Chemical composition of the investigated 17H1S steel (wt%).

Condition	Fe	C	Si	Mn	Cr	S	P
AR state	Balance	0.165	0.57	1.53	0.069	0.009	0.007
After LTO	Balance	0.171	0.47	1.25	0.028	0.0043	0.005
Regulated composition	Balance	0.15–0.2	0.4–0.6	1.15–1.6	<0.3	≤0.4	≤0.35

**Table 2:** Characteristics of strength (σ_UTS_ and σ_YS_), plasticity (*elongation at break*), and impact toughness (*KCV*) of the 17H1S main gas pipeline steel in the AR state and after LTO for 31 years.

Condition	Pipe Part (Marking)	Surface (Marking)	σ_UTS_, MPa	σ_YS_, MPa	Elongation at Break, %	*KCV*, MJ/m^2^
AR	-	Outer (I)	558	378	29.4	0.54
		Inner (II)	560	423	26.7	0.56
After LTO	Upper (T)	Outer (I)	500	291	28.6	0.36
	Lower (B)	Outer (IV)	545	325	27.6	-
	Upper (T)	Inner (II)	545	355	26.7	0.4
	Lower (B)	Inner (III)	544	351	26.7	0.32

## Data Availability

All the data are presented in the manuscript.
